# Estrogenic activity of food contact materials—evaluation of 20 chemicals using a yeast estrogen screen on HPTLC or 96-well plates

**DOI:** 10.1007/s00216-020-02701-w

**Published:** 2020-05-26

**Authors:** Alan J. Bergmann, Eszter Simon, Andrea Schifferli, Andreas Schönborn, Etiënne L. M. Vermeirssen

**Affiliations:** 1 Swiss Centre for Applied Ecotoxicology, Eawag, Überlandstrasse 133, 8600 Dübendorf, Switzerland; 2 Zürich University of Applied Sciences, Grüental 14, 8820 Wädenswil, Switzerland

**Keywords:** Endocrine-disrupting chemicals, Food packaging, Bioanalytics, Thin-layer chromatography, Dose-response modeling, Analysis of unknowns

## Abstract

Food contact materials (FCM) may contain complex mixtures of estrogenic chemicals. A yeast estrogen screen performed on high performance thin-layer chromatography plates (planar-YES, P-YES) is promising for analysis of such mixtures, as it could allow for better elucidation of effects compared with established methods in microtiter plates. However, the P-YES has not been directly compared with established methods. We compared the performance of a microtiter plate YES (lyticase-YES, L-YES) to P-YES on silica gel HPTLC plates using 17β-estradiol (E2), 20 chemicals representative of migrants from plastic FCM, and three migrates of coated metal food cans. Effective doses (ED_10_, ED_50_) and estradiol equivalencies were calculated for each chemical. Thirteen chemicals had calculable EDs in the L-YES or P-YES, with average EDs 13-fold (range 0.63–36) more potent in P-YES than in the L-YES. Normalized to E2, the median estrogenicity was within 1.5-fold (0.43–8.8) between the assays. Therefore, P-YES was as or more sensitive than L-YES but potencies relative to E2 were comparable between assays. With chromatography, the P-YES detected estrogenicity in coated metal cans, effects that were unmeasurable in L-YES. With the sample preparation methods used in this study, both YES assays are sufficiently sensitive to detect bisphenol A below the specific migration limit for plastic packaging (0.05 mg/kg food). This study demonstrates that P-YES outperforms L-YES because it is more sensitive, provides comparable estradiol equivalents, and circumvents confounding mixture effects. The P-YES will be useful for routine monitoring of FCM and toxicant identification in problematic materials.

**Electronic supplementary material:**

The online version of this article (10.1007/s00216-020-02701-w) contains supplementary material, which is available to authorized users.

**Figure:**
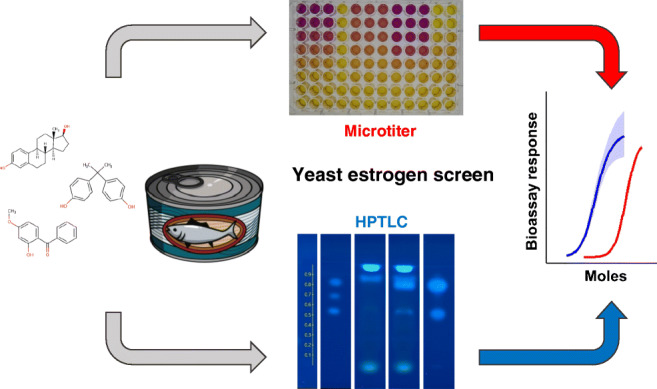
Graphical abstract

## Introduction

Food packaging can contribute potentially toxic chemicals to food. In the European Union, chemicals known to be reproductive toxicants are not allowed to be used in plastic food packaging [1], although some endocrine-disrupting chemicals are still associated with plastic packaging [2]. Food contact materials (FCM) used in packaging products can be a source of estrogenic chemicals to food [3]. Migration of known chemicals into food can be monitored with target chemical analysis. However, unintentional chemicals can also be present in the final packaging and may migrate to food. Therefore, methods to detect and identify toxic non-intentionally added substances (NIAS) are necessary beyond target analysis for known chemicals.

Bioassays are used to measure chemical mixtures for biological effects, so do not target specific chemicals. Bioassays for dioxin-like chemicals are allowed for screening of certain foodstuffs [4]. Bioassays are therefore potential tools to monitor for bioactive NIAS that can migrate from FCM [3, 5]. For samples with bioactivity, a combination of chemical fractionation and (bio)analysis, i.e., effect-directed analysis (EDA), is useful to elucidate the responsible toxicants [6, 7]. One important class of chemicals potentially coming from FCM is (xeno)estrogens, for which there are several types of bioassays. Commonly used are in vitro reporter gene assays that measure the activation of an estrogen receptor [8]. One such assay is the yeast estrogen screen (YES).

The YES employs yeast containing a plasmid with the human estrogen receptor, h-ERα, linked to *lacZ* gene that codes for β-galactosidase [9]. The response to a chemical or mixture is then measured with a substrate for β-galactosidase whose product results in a colored or fluorescent signal. A standardized 96-well plate format of the YES performed with enzyme (lyticase)-assisted digestion is referred to here as the L-YES [10]. In such reporter gene assays, mixture components can cause cytotoxicity which can mask transactivation [5]. To handle interfering cytotoxicity, Escher et al. recommend running parallel cell viability assays and limiting dose-response modeling to low, non-cytotoxic concentrations [11]. This is successful if cytotoxicity only occurs at concentrations greater than observable target effects, e.g., h-ERα induction.

Bioassays are also possible on high performance thin-layer chromatography (HPTLC) plates. Such methods may first perform chromatographic separation of a chemical mixture using TLC. Then, an in vitro bioassay is performed on the HPTLC plate where it can be possible to distinguish target effects from cytotoxicity [12–15]. This combination of sample separation and bioassay is well suited to an EDA framework [6, 12, 16]. For example, the retention factor of bioactive zones can be matched with those of known chemicals to help identify responsible toxicants, and active zones can be extracted for further evaluation with mass spectrometry. The yeast estrogen screen is one of the most established assays in HPTLC format [12–14, 17, 18]. Referred to here as planar-YES (P-YES, and specifying with or without chromatography), it has been used for analyses including wastewater [14, 15], river sediment [14], herbal extracts [19], and personal care products [12], but until recently is still undergoing development in data collection [17, 18] and evaluation [20, 21].

Previous work comparing P-YES to other assays has focused on steroidal estrogens. Könemann et al. found that results of P-YES correlated well to human cell-based transactivation assays for surface and wastewater samples [22], although a 96-well plate YES was not included in their comparisons. The study focused on three steroidal estrogens commonly detected in surface and waste water samples, for which predicted effects (based on chemical analysis of three target estrogens) matched the bioassay results well. Klingelhöfer and Morlock also compared the potency (relative to 17β-estradiol, E2) of 17α-ethinyl estradiol in P-YES to literature values of a microtiter well plate version of YES with good agreement (0.3 to 0.47, respectively) [18]. It is still unknown how P-YES differs from microtiter plate assays for a broader range of chemicals, specifically those that are relevant to FCM. Differences in assay details, such as silica or polystyrene plate material, could have chemical-specific effects on bioassay results [23].

With the aim of further establishing P-YES for bioassay screening and EDA of FCM, this study compares the P-YES to a 96-well plate-based L-YES. Our specific goals were to (1) define the relative sensitivities of P-YES and L-YES to several estrogenic chemicals related to FCM, (2) understand any discrepancies between the assays by evaluating assay parameters, and (3) evaluate the performance of these two assays for migrates from real FCM. Results from this work will help to establish the P-YES as a tool for analysis of single chemicals and complex mixtures such as FCM.

## Materials and methods

### Materials

HPLC-grade ethanol, methanol, *n*-hexane, acetone, petroleum ether, chloroform, and silica gel 60 HPTLC plates were purchased from Merck (Darmstadt, Germany). Lyticase, dithiothreitol, chlorophenol red-β-d-galactopyranoside (CPRG), and 4-methylumbelliferyl-β-d-galactopyranoside (MUG) were obtained from Sigma-Aldrich (St. Louis, Mo, USA). A solution (*lacZ* buffer) was prepared with 10.67 g/L Na_2_HPO_4_-2H_2_O, 0.75 g/L KCl, 0.25 g/L MgSO_4_-7H_2_0, and 1 g/L sodium dodecyl sulfate (all from Sigma-Aldrich). We selected chemicals for screening based on their reported presence in food packaging materials and for their potential estrogenicity [24–26]. Table 1 lists the test chemicals, and their sources and purity, which include four phenols, three bisphenols, three phthalates, and two benzophenones.

**Table 1:** Test substance physicochemical properties^a^

Substance	CAS	Source, purity	Average mass (g/mol)^*a*^	Henry’s law (atm-m^3^/mol) ^*a*^	LogK_OA_^a^	LogK_OW_^a^	Water solubility (mol/L)^a^
Diethylhexyl adipate	103-23-1	Sigma, 99%	370.6	3.92 × 10^−7^	10.86	6.85	3.90 × 10^−6^
4-Nonylphenol	104-40-5	Alfa Aesar, > 98%	220.4	1.06 × 10^−5^	9.36	5.67	3.64 × 10^−5^
Benzene, 1,1′-(1,3-propanediyl)bis-	1081-75-0	TCI, > 95%	196.3	2.04 × 10^−4^	7.43	3.95	1.21 × 10^−5^
Phenol, 4-cyclohexyl-	1131-60-8	TCI, > 98%	176.3	7.18 × 10^−7^	8.66	4.10	8.97 × 10^−4^
Triphenyl phosphate	115-86-6	TCI, > 99%	326.3	1.78 × 10^−6^	10.80	4.59	5.70 × 10^−6^
Butylated hydroxytoluene	128-37-0	Sigma, 99%	220.4	7.99 × 10^−6^	9.29	5.05	6.15 × 10^−6^
2,2′-Dihydroxy-4-methoxybenzophenone	131-53-3	TCI, > 98%	244.2	8.51 × 10^−10^	8.40	2.80	1.10 × 10^−4^
2-Hydroxy-4-methoxybenzophenone	131-57-7	Sigma, 98%	228.2	1.05 × 10^−9^	9.72	3.36	2.10 × 10^−4^
Nonylphenol ethoxylate	26027-38-3	Sigma	–	–	–	4.48	–
Nonylphenyl phosphite (3:1)	26523-78-4	Sigma	–	–	–	–	–
2,4-Bis(1-methyl-1-phenylethyl)phenol	2772-45-4	TCI, > 97%	330.5	3.11 × 10^−7^	10.54	6.45	1.62 × 10^−5^
Bis(4-hydroxyphenyl)methane	620-92-8	Sigma, > 98%	200.2	1.25 × 10^−7^	8.18	3.14	2.36 × 10^−3^
Bisphenol B	77-40-7	TCI, > 98%	242.3	2.71 × 10^−7^	8.99	3.77	4.97 × 10^−4^
Bisphenol A	80-05-7	Sigma, > 99%	228.3	1.26 × 10^−7^	8.38	3.35	5.44 × 10^−4^
Diisobutyl phthalate	84-69-5	Sigma, > 99%	278.3	3.13 × 10^−7^	8.21	4.28	2.90 × 10^−5^
Di-*n*-hexyl phthalate	84-75-3	TCI, > 98%	334.5	3.42 × 10^−8^	9.67	6.17	2.24 × 10^−7^
Butyl benzyl phthalate	85-68-7	Sigma, > 98%	312.4	2.82 × 10^−8^	9.83	4.31	9.39 × 10^−6^
4-*tert*-Butylphenyl salicylate	87-18-3	TCI, 98%	270.3	4.57 × 10^−10^	10.24	3.96	1.30 × 10^−4^
4-Phenylphenol	92-69-3	Sigma, 99%	170.2	1.04 × 10^−7^	9.20	3.26	6.71 × 10^−4^
2,4-Di-*tert*-butylphenol	96-76-4	Sigma, > 99%	206.3	9.36 × 10^−6^	8.67	4.97	6.11 × 10^−5^

^a^Values from Chemistry Dashboard (U.S. EPA) accessed 18.10.2018. OPERA-predicted values used for Henry's Law, *logK*_*OA*_ log octanol-air partitioning coefficient, *logK*_*OW*_ log octanol-water partitioning coefficient, and water solubility

### Estrogen screening

Yeast (*S. cerevisiae* of “McDonnell” [27] or “Sumpter” [28] strains) was stored as concentrated stock cultures, at 900–1400 formazine attenuation units (FAU, a measure of cell density), in 20% glycerol at − 80 °C until bioassays were performed. Yeast stock cultures were used to inoculate growth media 22 ± 1 h before starting the bioassays. Cell density was measured with a microtiter plate absorbance reader (BioTek Synergy 2, Winooski, VT, USA) at 600 nm. Yeast were prepared for L-YES and P-YES in exposure media according to an ISO standard [10] and Schönborn et al. [17].

#### L-YES

The L-YES procedure followed an ISO standard [10] in 96-well plates. Ethanolic solutions of test chemicals were serial diluted over eight wells, then evaporated to dryness. Eighty microliters of nanopure water was added to each well and shaken for 5 min at 500 rpm. Then, 40 μL of yeast at a density of 25 FAU was added to each well; the plates were covered with breathable seals and incubated at 30 °C for 18 ± 1 h. After incubation, the cells were re-suspended and cell density was recorded. Aliquots (30 μL) of yeast were transferred from the wells of the test plate to a new 96-well plate and 50 μL of 0.4 mg/mL CPRG solution with 250 U/mL lyticase and 1 mM dithiothreitol in *lacZ* buffer was added to each well. After an incubation of 1 h at 30 °C, absorbance was measured at 580 nm.

#### High performance thin-layer plate preparation and chromatography

HPTLC plates (20 × 10 cm, or 10 × 10 cm in some tests without chromatography) were prepared by developing them with methanol in a twin-trough chamber then drying in an oven at 110 °C for 30 min. Chemicals, prepared in ethanol, were applied in up to 20 μL per band to HPTLC plates with an Automated TLC Sampler 4 (CAMAG, Muttenz, CH) as 6 mm bands starting 20 mm from the left edge, and track distance at least 18 mm. Additional plate layout and application parameters are given in Electronic Supplementary Material (ESM) Tables S1 and S2. For tests without chromatography, application of chemicals was randomly assigned along three rows at 15, 42.5, and 70 mm on each plate. We used chromatography only in experiments to compare its effect on the potency of E2, the reference chemical for both YES assays and bisphenol A (BPA), as described in the ESM (Fig. S8), and when testing migrates of FCM. In these cases, HPTLC plates were developed to 80 mm with an Automated Multiple Development 2 (AMD2, CAMAG) using preconditioning with 1.2% NH_3_, isocratic chromatography with a solvent mixture of chloroform:acetone:petroleum ether 11:5:5 [17, 29], and dried under vacuum for 2 min. For experiments described in this study, P-YES was performed without chromatography unless otherwise specified (as “P-YES with chromatography”).

#### P-YES

Performance of the P-YES was based on Schönborn et al. [17]. Yeast was adjusted to 1000 ± 200 FAU in fresh exposure medium. With an automated spraying chamber, Derivatizer (CAMAG) with red nozzle and spraying level six, 2 mL of yeast culture was sprayed onto HPTLC plates before or after chromatography. The HPTLC plates were incubated for 3 h at 30 °C in plastic boxes with water-saturated paper towels to maintain humidity above 80%. After incubation, the plates were dried with a hair dryer set on low heat and fan speed for 3–5 min. The indicator, 2 mL 0.5 mg/mL MUG in *lacZ* buffer, was then sprayed onto the plates with the Derivatizer (blue nozzle, level six) and plates were incubated at 37 °C for 20 min. The plates were dried again with a hair dryer. For cases in which fluorescent signal on a plate was uniformly less than expected, plates were exposed to NH_3_ vapor, which enhanced the signal of the fluorescent product of MUG, 4-methylumbelliferone (4-MU) [13, 30]. Images were collected with the TLC Visualizer (CAMAG) with illumination at 366 nm for 550 ms and processed for peak height and area with VisionCats v2.4 (CAMAG).

### Chemical testing

Twenty chemicals were evaluated in duplicate in 10-fold dilution series for range-finding in L-YES and P-YES. The highest concentrations were 1 mM in L-YES. The moles of chemical in 1 mM in L-YES (final volume 120 μL) were applied as the highest level in P-YES. Fourteen chemicals were determined active in range-finding tests. To capture the full dose-response curves, these chemicals were tested again in the L-YES and P-YES with 2-fold dilutions in suitable concentration ranges (ESM Table S3) based on the range-finding tests.

The effect of chromatography on sensitivity of the assays was evaluated for E2 and BPA. Briefly, E2 and BPA concentration series were applied, with randomized tracks, to part of an HPTLC plate, and developed in the AMD2. Then, the chemicals were applied in randomized tracks to the remaining part of the HPTLC plate. The P-YES proceeded on the HPTLC plates as described above, and analyzed as described below.

### Sample preparation

Food contact articles were donated by the Cantonal Laboratory of Zurich and Swiss Quality Testing Services (SQTS). All samples were metal cans with internal coating. From SQTS, a fish can was selected because it had previously displayed estrogenic activity in P-YES with chromatography [31]. Duplicate cans received 95% ethanol:water and were sealed with the original lids and PTFE tape (Sigma-Aldrich), with no internal standards added. The duration of the migrations lasted for 10 days at 60 °C. Two glass beakers, sealed and handled in the same way, were used as migration controls. Food can migrates were concentrated 20-fold by evaporation with constant stream of nitrogen in a 40 °C water bath using Turbovap LV (Biotage, Uppsala, SWE), to a final volume of 1 mL. They were tested up to 2.4 mL migrate equivalents (120 μL concentrated migrate) in L-YES and up to 0.8 mL migrate equivalents (40 μL concentrated migrate) in P-YES with chromatography. The bioassay and detection were performed as described above.

### Quality control and data analysis

The potency of E2 was monitored over time and was stable for experiments included in this study (see ESM Figs. S1 and S2). Solvent and migration controls were inactive in both bioassays. The format of P-YES (chemical application to HPTLC plates and solvent evaporation) prohibits calculation of a concentration-based dose. Therefore, for both assays, the number of moles per replicate, i.e., moles per application zone or moles per well in P-YES and L-YES respectively, was used as a dose metric.

Dose-response modeling of P-YES results was performed with R using the package *drc* 3.0-1 [32]. P-YES dose-response modeling consisted of the following. Peak height of E2 was modeled as a 4-parameter log logistic regression (Eq. 1) for each replicate plate. Then, height of E2 and test chemicals was normalized to the modeled top E2 response for each plate. Replicates were combined and modeled again with Bottom constrained to zero (Eq. 2). Based on examination of model residuals, logarithm of response was used to comply with the modeling assumption of constant variance, and a constant was added to allow log transformation of zero values. Absorbance at 580 nm measured in triplicate wells in the L-YES was corrected for the average absorbance of ethanol controls. The data were normalized to the modeled top E2 value (Eq. 1) and modeled with Eq. 2, without log-transformed response (GraphPad Prism 7, San Diego, CA, USA). Treatments that caused less than 80% of the average cell growth of ethanol controls, as measured with absorbance at 600 nm, were omitted from analysis.

1 $$ \mathrm{height}=\mathrm{bottom}+\frac{\mathrm{top}-\mathrm{bottom}}{1+{e}^{\left(\ \log \mathrm{mole}-\log {\mathrm{ED}}_{50}\right)\times \mathrm{hillslope}}} $$

2 $$ \log \left(\mathrm{normalized}\ \mathrm{height}+1\right)=0+\frac{\mathrm{top}-0}{1+{e}^{\left(\ \log \mathrm{mole}-\log \left(\mathrm{inflection}\right)\right)\times \mathrm{hillslope}}} $$

Median or 10% effective doses (ED_50_ or ED_10_, respectively) and 95% confidence intervals were determined from interpolation of dose-response curves at 50 and 10% of normalized E2, e.g., at y = log(50 + 1) using Eq. 2. Estradiol equivalency factors (EEFs) were determined when possible as the ratio of ED_50_s of E2 to corresponding test chemicals for each experiment (Eq. 3). ED_10_ was used to calculate chemical EEF when ED_50_ from at least one bioassay was incalculable, e.g., due to low maximum response.

3 $$ \mathrm{EEF}\ \left(\mathrm{unitless}\right)=\frac{{\mathrm{ED}}_{50\ \mathrm{E}2}\left(\mathrm{mole}\right)}{{\mathrm{ED}}_{50\ \mathrm{test}\ \mathrm{chemical}}\left(\mathrm{mole}\right)} $$

## Results and discussion

### P-YES is more sensitive than L-YES

**Fig. 1:**
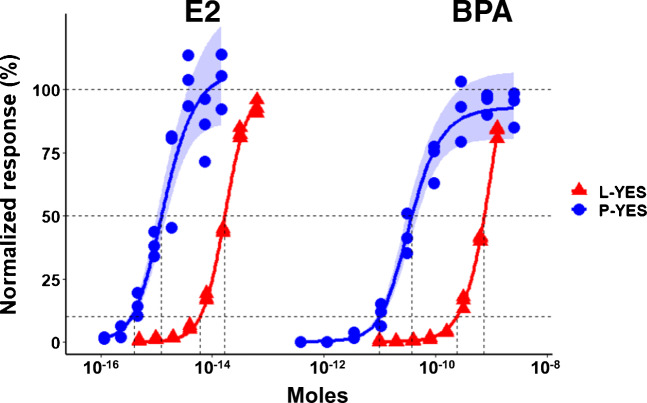
Example dose-response curves used in the calculation of ED_50_ and ED_10_, which are indicated by vertical dashed lines. The responses are peak height for P-YES and optical density at 580 nm for L-YES. Replicates are three HPTLC plates (one replicate concentration series per plate) or three wells of a microtiter plate (three replicate concentration series per plate) for P-YES and L-YES, respectively. E2, 17β-estradiol; BPA, bisphenol A

Based on previous publications [17, 33] and our own preliminary work, the most sensitive methods, i.e., appropriate yeast strain, for performing L-YES and P-YES were used to analyze chemicals associated with FCMs. The reference compound, E2, was more potent in P-YES than L-YES by about 10-fold (Fig. 1 and ESM Fig. S2).

**Fig. 2:**
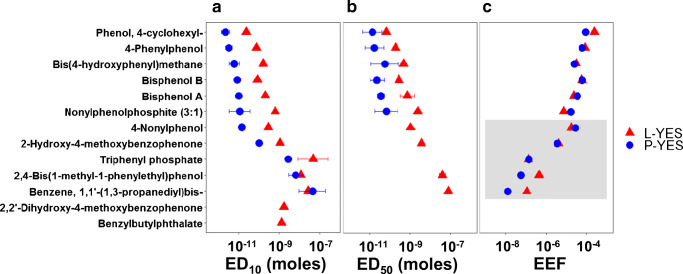
Estrogenicity of chemicals related to FCM in the L-YES and P-YES. (a) Ten percent, and (b) median, effect concentrations (ED_10_ and ED_50_, respectively) with 95% confidence intervals (*n* = 3–4). Error bars are often hidden by the data marker. (c) Relative potency as normalized to the reference compound E2 (17β-estradiol equivalency factor, EEF). Center values are shown for only chemicals for which EEFs in both assays could be calculated. EEFs in gray shading were calculated with ED_10_ instead of ED_50_ for chemicals that did not reach 50% effect level

Thirteen chemicals had calculable effect concentrations for at least the 10% level in either L-YES or P-YES (Fig. 2, ESM Table S4). Full dose-response curves are shown in ESM Fig. S3. Diisobutyl phthalate, which is not shown in Fig. 2, showed slight induction in the L-YES but below ED_10_. An ED_10_ was calculable from both assays for 11 chemicals, and an ED50 was calculable in both assays for six chemicals. Benzyl butyl phthalate and 2,2′-dihydroxy-4-methoxybenzophenone had poor responses in L-YES, with a maximum induction of less than 50% of the E2 maximum.

The active chemicals were on average 13-fold (range 0.63–36) more potent in P-YES than L-YES. Previous testing of single chemicals showed similar findings in yeast-based assays. Our determined ED_50_s of 2,4-bis(1-methyl-1-phenylethyl) phenol and benzene, 1,1′-(1,3-propanediyl)bis- in the L-YES were very similar to previous values determined in a YES performed in microtiter plates, while the P-YES produced EDs closer to those determined with ERα-CALUX [34]. Harris et al. found an EEF of 1 × 10^−6^ and 1 × 10^−7^ for benyzl butyl phthalate and diisobutyl phthalate, respectively [35]. In comparison, we found the L-YES EEF for butyl benzyl phthalate to be about 5 × 10^−6^, and the modeled top values of these phthalates were 50% or less of the modeled E2 top, as has also been previously observed [35, 36]. Compounds that were inactive in both L-YES and P-YES also corroborate previous findings, for example, 2,4-tert-butylphenol, butylated hydroxytoluene, and diethylhexyl adipate were not active at concentrations tested by Mertl et al. [34] or Simon et al. [37].

The variability of the P-YES was always greater than the L-YES. The dose-response curves of E2 and BPA had larger confidence intervals in the P-YES than the L-YES (Fig. 1). The variability of P-YES results is also apparent for the other test chemicals in the ED_10_ and ED_50_ confidence intervals (Fig. 2) and full dose-response models (ESM Fig. S3). However, we challenged the P-YES by using three HPTLC plates as replicates compared with the ISO L-YES, in which replicates were on the same microtiter plate. In this way, some, but probably not all, of the variability differences can be explained.

### EEFs are similar despite different assay formats

**Fig. 3:**
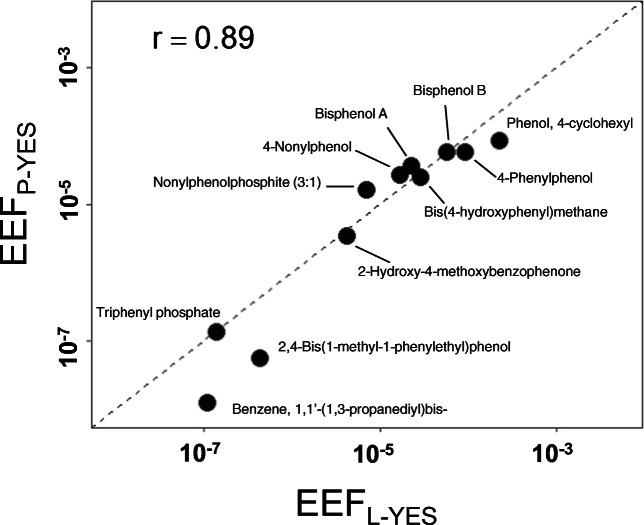
Estradiol equivalency factors (EEF) for chemicals that tested positive in the L-YES and P-YES. Dashed line represents 1:1. Based on EDs as described in Fig. 2. *P* value for Pearson correlation (*r*) = 0.00031

In general, we observed good agreement in EEF between L-YES and P-YES. The assays produced similar results when normalized to the reference chemical, E2, as EEF (Fig. 2c). The median EEF was 1.2 (range 0.43–8.8) times greater in the L-YES than for P-YES. The Pearson correlation of EEFs between the P-YES and L-YES was 0.89 (Fig. 3). This suggests that differences between tested chemicals and assay format do not have a big effect on quantification of estrogenicity. This is surprising as the availability to yeast cells could depend on the plate materials and format of the bioassay. Both the microtiter and HPTLC plate assays include a similar step in which test chemicals and samples are applied to a plate in organic solvent and dried. The test substance is then re-dissolved in water or medium when a solution is added to the microtiter wells or the yeast suspension is sprayed onto the HPTLC plate, respectively. However, plate materials (polystyrene in L-YES, silica in P-YES) have different affinities for test chemicals which could affect their availability in exposure medium [38]. The clearest trend in chemical responses was decreasing agreement in EEF between L-YES and P-YES with decreasing potency (Fig. 2). Effect concentrations were not easily modeled for chemicals with lower potency due to low maximum responses (e.g., benzyl butyl phthalate) or reduced cell growth (e.g., 2,2′-dihydroxy-4-methoxybenzophenone). Correlation between estrogenic activity and Henry’s law, octanol-air, or octanol-water coefficients was poor (Pearson’s correlation ≤ 0.5). A larger suite of chemicals might be able to elucidate subtle differences between assays. However, we did notice that the agreement between P-YES and L-YES seems to hold until potency approaches water solubility in the L-YES (ESM Fig. S4). Water solubility in P-YES is not calculable because the volume in which chemicals are dissolved is unknown. A specific case is for 2,2′-dihydroxy-4-methoxybenzophenone which has similar water solubility and L-YES potency as 2-hydroxy-4-methoxybenzophenone but did not produce a response at 10% effect in P-YES. This is possibly explained by greater inhibitory effects (halo in P-YES or reduced cell growth in L-YES) of 2,2′-dihydroxy-4-methoxybenzophenone than 2-hydroxy-4-methoxybenzophenone. Overall, while P-YES and L-YES show good consensus, the agreement might break down as one or both assays reach physical limits such as water solubility.

### Many assay parameters do not explain different sensitivities

This study used two different yeast strains for the P-YES (“McDonnell”) [27] and L-YES (“Sumpter”) [28]. This difference was based on established protocols [10, 17], and we investigated the performance of the strains in both assays. The ED_50_ of the reference chemical, E2, was lower for Sumpter than McDonnell yeast in the L-YES. The background response was also lower for Sumpter yeast than McDonnell (ESM, Fig. S5). Both strains produced similar responses to 20 food contact chemicals tested in the L-YES (ESM, Fig. S6). Therefore, the strains do not have major inherent differences in their response to different chemicals. In the P-YES, Sumpter yeast produced a much weaker response than McDonnell, so was not used to evaluate the whole suite of chemicals. Sumpter yeast requires media enriched with more amino acids than McDonnell. It is possible that nutrients are not as available on HPTLC plates as when dissolved in wells of a microtiter plate. This would allow the more self-sufficient McDonnell yeast to thrive better than Sumpter yeast on HPTLC plates.

As mentioned in the previous section, some chemicals produced a “halo” or “corona” effect in P-YES (see ESM, Fig. S7). The halo is characterized by a ring of fluorescence around an inactive zone (or activity below baseline). This has been attributed to cytotoxicity [12], and to fluorescence quenching [39]. The two benzophenones were among those that produced halos in P-YES. Benzophenones are used as blockers of ultraviolet (UV) light in consumer products, and other chemicals naturally fluoresce under UV. Because detection of 4-MU, the fluorescent product in the P-YES, requires illumination at 366 nm, these chemical properties may interfere with the results of the test. Therefore, the benzophenones were also tested with CPRG as an indicator solution. The same degree of halo was observed with CPRG as with MUG (e.g., ESM, Fig. S7) so there was no obvious effect of UV blocking causing the differences observed between P-YES and L-YES. Müller et al. observed a 20-fold smaller lowest observed effect level with MUG than CPRG as indicators for YES on HPTLC plates [13]. To test how these indicators affect sensitivity, the L-YES was performed with MUG as well as CPRG. The ED_50_ of E2 was an average of 1.3 (SD = 0.35, *n* = 3) times lower for a 1 h incubation with MUG or CPRG (ESM Table S5). Thus, indicator solution alone does not explain the differences observed for greater sensitivity in the P-YES.

Samples, including migrates from FCM, can contain chemicals that have native fluorescence at wavelengths that could interfere with MUG fluorescence. With HPTLC, estrogenic chemicals can be separated from native fluorescence, elucidating bioactivity in a sample that might be hidden when testing the whole sample, as with the L-YES. Samples can also have color which could affect measurement with CPRG. An alternative is resorufin-β-d-galactopyranoside, producing an orange fluorescent signal, which has been used on HPTLC plates to detect E2 down to 3.5 pg/zone (1.3 × 10^−14^ mol/zone) [21].

Chromatography, which is used in most cases before bioassays on HPTLC plates, may affect the quantitative results, for example, through diffusion of chemicals into the plate. Because this study focuses on effects of the bioassay format (microtiter vs HPTLC plates) between L-YES and P-YES, most experiments were done without chromatography. We also evaluated the effect of one chromatography method on quantitation of E2 and BPA as model chemicals (described in ESM for Fig. S8). The ED_50_s of E2 and BPA (moles with 95% confidence interval) were 1.1 × 10^−15^ (9.1 × 10^−16^–1.4 × 10^−15^) and 2.0 × 10^−11^ (1.5 × 10^−11^–2.6 × 10^−11^) without chromatography (P-YES), and 2.0 × 10^−15^ (1.7 × 10^−15^–2.4 × 10^−15^) and 2.5 × 10^−11^ (1.9 × 10^−11^–3.3 × 10^−11^) with chromatography. In this direct comparison, the confidence intervals for E2 with and without chromatography do not overlap, suggesting a significant difference. However, both ED_50_s, E2 with and without chromatography, are within the variability of values measured in the rest of this study (ESM Figs. S1 and S8) and consistently lower than the E2 ED_50_ in L-YES. Spira et al. [14] and Riegraf et al. [40] observed higher effective doses with chromatography than without, for a P-YES and an HPTLC-based algae bioassay, respectively. These studies used similar, isocratic, chromatography methods as in the current work. They differed, however, in the application method of cells, test species, and/or measurement of effect, any of which might contribute to seeing effects of chromatography [14, 40]. In this study, we intended to focus on the intrinsic differences between the microtiter and HPTLC plate formats of the two yeast assays. There may be combinations of target analyte, sample matrix, chromatography, and bioassay detection that affect the sensitivity of an HPTLC bioassay. Our results show that the P-YES is intrinsically more sensitive than the L-YES, and chromatography will not universally reduce the sensitivity of the bioassay.

### Quantifying with area instead of height does not affect the test outcome

To comply with modeling assumptions, we evaluated P-YES data in this study using peak height [20]. However, area under the curve might be more appropriate to compare chemicals if they have different peak shapes. Therefore, we investigated the effect of using area on this study’s outcome. Possibly from diffusion of chemicals on HPTLC plates, we observed continuously increasing areas which is a problem for defining top values of the reference compound E2. Others have observed the same, including on reverse phase-wettable HPTLC plates and with multiple yeast densities [20]. This also leads to violation of modeling assumptions of constant variance. Although if we ignore the modeling violations, the same conclusions are reached with area as with height: P-YES is more sensitive but yields similar EEF when compared with L-YES (ESM Fig. S9).

### P-YES reveals estrogenicity of food contact materials where L-YES does not

**Fig. 4:**
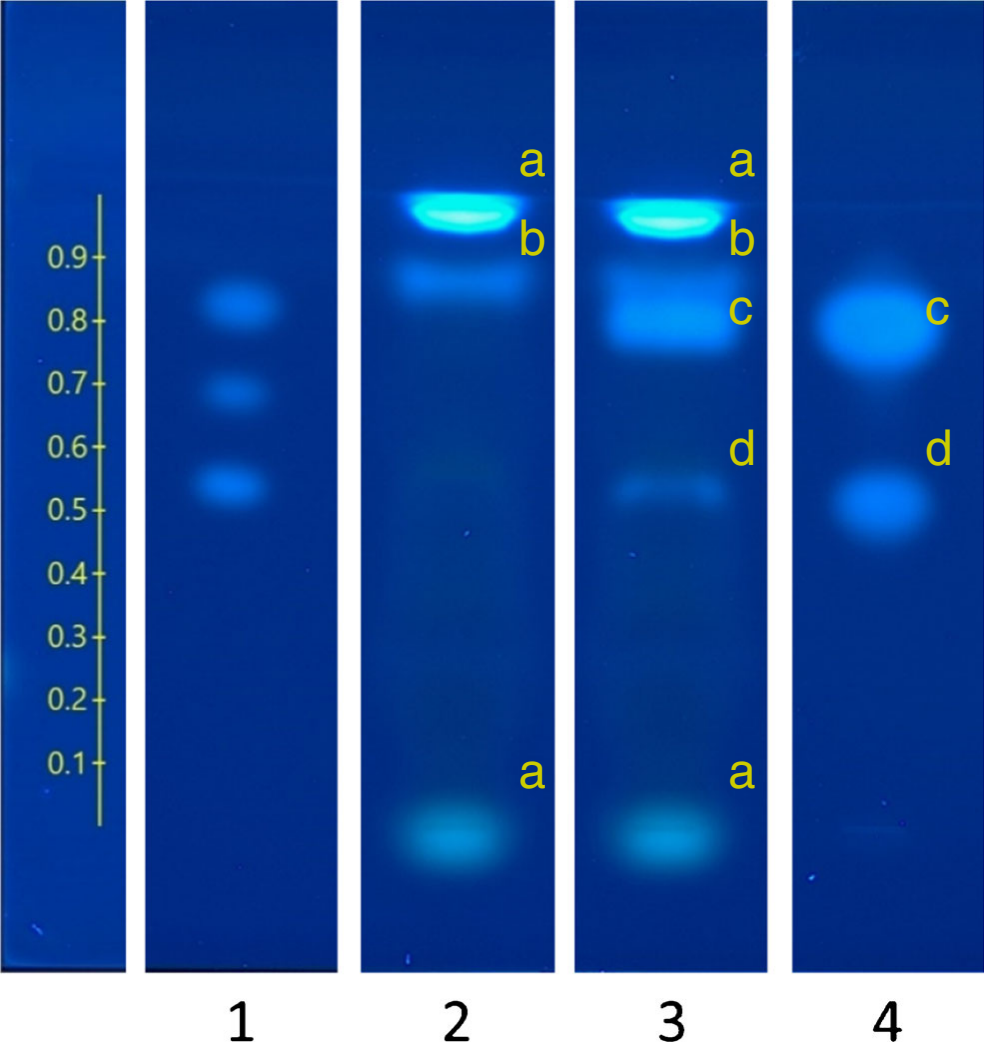
Detection of native and spiked (xeno)estrogens in a migrate of lined metal can (fish can). Lane 1: positive control mixture of (bottom to top) 17β-estradiol (1 pg), 17α-ethinyl estradiol (1 pg), and estrone (10 pg). Retention factor shown on the left. Lane 2: native fish can migrate, 0.4 mL migrate equivalents. Lane 3: fish can migrate spiked with three (xeno)estrogens. Lane 4: Control migrate spiked with three (xeno)estrogens. Zones marked with (a) native fluorescence of chemicals (i.e., not estrogenicity), (b) native estrogenicity, (c) co-retained spiked chemicals estrone (0.2 ng) and 2-hydroxy-4-methoxybenzophenone (140 ng), (d) bisphenol A (27 ng)

Three migrates of coated metal cans were evaluated in the L-YES and P-YES with chromatography. Images of P-YES with chromatography for the sample with the strongest response (fish can) are highlighted in Fig. 4, showing bands of native (unknown) and spiked (xeno)estrogen. In L-YES, these migrates produced only slight induction (below 10% of the maximum E2 response), if at all. The highest concentrations of two samples resulted in reduced cell density compared with controls (ESM Fig. S10 and Fig. S11). The same samples showed zones of estrogenicity in P-YES with chromatography (ESM Fig. S12). Fish can migrates showed a strong induction in a band near the solvent front. Therefore, we conclude that the detection of xenoestrogens in the fish can was obscured by low cell growth in the L-YES.

We have not determined the identity(ies) of the estrogenic substance(s) in the migrates. Although not the purpose of this study, we could further investigate toxicants detected with P-YES by extracting them for chemical analysis such as non-target high-resolution mass spectrometry [41]. Then, retention factors of standards (if available) from P-YES with chromatography can be used for confirmation. Toxicant identification can be time and resource intensive in the effort to determine the structure of one unknown estrogenic chemical [6]. An alternative, if the chemical’s identity remains unknown, is to quantify the bioassay effect relative to a reference compound. Bioassays to determine bioanalytical equivalents are allowed in determination of dioxin-like activity in food [4]. Similarly, estradiol equivalencies (EEQs) have been applied toward threshold-based screening of water samples [42]. EEQs derived using a version of P-YES with chromatography were shown to correlate well with EEQs from human cell line assays [22].

The specific migration limit for BPA was recently lowered from 0.6 to 0.05 mg/kg food [43]. We used the migration conditions for the fish cans in this study to determine if the L-YES and P-YES are sufficiently sensitive to detect BPA at the updated limit. We assumed a contact ratio of 6 dm^2^/kg food [1] and used 1.9 dm^2^ as the estimated internal surface area of the cans, which contacted 80 mL of solvent during migration. Using these assumptions, we calculated a necessary detection limit of BPA as 0.2 mg/L migrate. When we applied 0.8 mL migrate equivalents to the HPTLC plates (40 μL of 20× concentrated migrate) and assuming 100% recovery during sample preparation, we needed to therefore be able to detect 0.2 μg (9 × 10^−10^ mol) in the bioassay. Both L-YES (BPA ED_10_, 2.0 × 10^−10^ mol) and P-YES (BPA ED_10_, 9.8 × 10^−12^ mol) are sufficiently sensitive to detect BPA under these conditions. In addition, we demonstrate that BPA was detected when spiked in the fish can migrate at a level of 1.2 × 10^−10^ mol (27 ng) per band (Fig. 4). However, it should be considered that sample matrix could impact band shape, ultimately affecting quantitation.

## Conclusions and recommendations

The P-YES was more sensitive than the L-YES, and is capable of revealing estrogenic effects which might be concealed when testing the whole complex mixture, as in the L-YES. The L-YES was more precise than the P-YES in measuring the dose-response curves of individual chemicals, but both assays produced similar relative potencies, EEFs. We expect these findings will be relevant for other in vitro bioassays performed on HPTLC plates. However, with the relatively small number of test chemicals, range of physicochemical parameters occupied by estrogens, and the variability in methods used for HPTLC bioassays, more data will be needed to confirm our results in other tests and analyte combinations. The L-YES may be easier to implement for laboratories already performing microtiter assays and little experience with HPTLC. Standardization of HPTLC bioassay methods would support transferability of P-YES methods. Our aim was not an easy assay but one that reveals toxicity in complex mixtures. For this, and greater sensitivity, our experience demonstrates that HPTLC bioassays are worth considering for incorporation into routine analysis of NIAS in FCM and other complex mixtures.

## Electronic supplementary material

ESM 1 (PDF 967 kb)

## Data Availability

The datasets generated during the current study are available from the corresponding author on request, and will be available at https://opendata.eawag.ch/.
