# Supplementary material for: Estrogenic activity of food contact materials—evaluation of 20 chemicals using a yeast estrogen screen on HPTLC or 96-well plates
Source: Anal Bioanal Chem. 2020 May 26;412(19):4527–36. doi: 10.1007/s00216-020-02701-w (PMC7329773; doi:10.1007/s00216-020-02701-w)
Supplement: Supplementary file 1 — (PDF 967 kb) [file 216_2020_2701_MOESM1_ESM.pdf]

Analytical and Bioanalytical Chemistry

Electronic Supplementary Material

Estrogenic activity of food contact materials – evaluation of 20 chemicals using a yeast estrogen screen on HPTLC or 96-well plates

Alan Bergmann, Eszter Simon, Andrea Schifferli, Andreas Schönborn, Etienne Vermeirssen

List of figures

Figure S1. Reproducibility of reference chemical, E2, in P-YES 4
Figure S2. Median effective doses of E2 in P-YES compared to L-YES 4
Figure S3. Full dose-response curves of individual chemicals....................13
Figure S4. Dose at 10% effect compared to water solubility 14
Figure S5. Examples of McDonnell and Sumpter strains of yeast 15
Figure S6. Comparison of ED_{50}s derived from McDonnell yeast in L-YES with Sumpter yeast in L-YES and McDonnell yeast in P-YES 15
Figure S7. Example of “halo” or “corona” effect in P-YES....................16
Figure S8. Investigating the effect of chromatography 17
Figure S9. Effective doses and estradiol equivalencies calculated with P-YES peak area....................18
Figure S10. Fish can migrate tested in L-YES 18
Figure S11. Cell density in L-YES of fish can migrate....................19
Figure S12. Estrogen screening of food contact materials with P-YES 19

List of tables

Table S1. “Plate Layout” settings for VisionCats Analyses 3
Table S2. “ATS4 application” settings for VisionCats Analyses 3
Table S3. Range of doses (moles) applied in full dose-response curves of test chemicals....................5
Table S4. Effective doses and their 95% confidence intervals 6
Table S5. L-YES with CPRG and MUG 16

Table S1 “Plate Layout” settings for VisionCats Analyses used for screening chemicals without chromatography on HPTLC plates

**Parameter**	**Setting**
Plate manufacturer	Merck
Stationary phase	HPTLC plates silica gel 60
Width	200 mm
Height	100 mm
Application position	15, 42.5, or 70 mm
First track position	20 mm
Track distance	18 mm
Application length	6 mm
Application width	0 mm
Solvent front position	90 mm

Note: A different application “Analysis” was used for each row and the final analysis. The solvent front position was set to a value greater than the application position for the “Analysis” programs.

Table S2 “ATS4 application” settings for VisionCats Analyses used for screening chemicals without chromatography on HPTLC plates

**parameter**	**setting**
Dosage speed	120 nL/s
Predosage volume	200 nL
Filling speed	11 µL/s
Filing vacuum time	1 s
Retraction volume	200 nL
Rinsing vacuum time	6 s
Rinsing cycles	2
Do not rinse before next vial	Not checked
Empty syringe before filling	Not checked
Flush nozzle	Checked
Filling cycles	1
Fill only programmed volume	Not checked
Return unused sample into vial	Checked
Syringe volume	25 µL
Spray gas	N_2
Rack type	Standard
Vial bottom level	0.0
Nozzle heating	Not checked

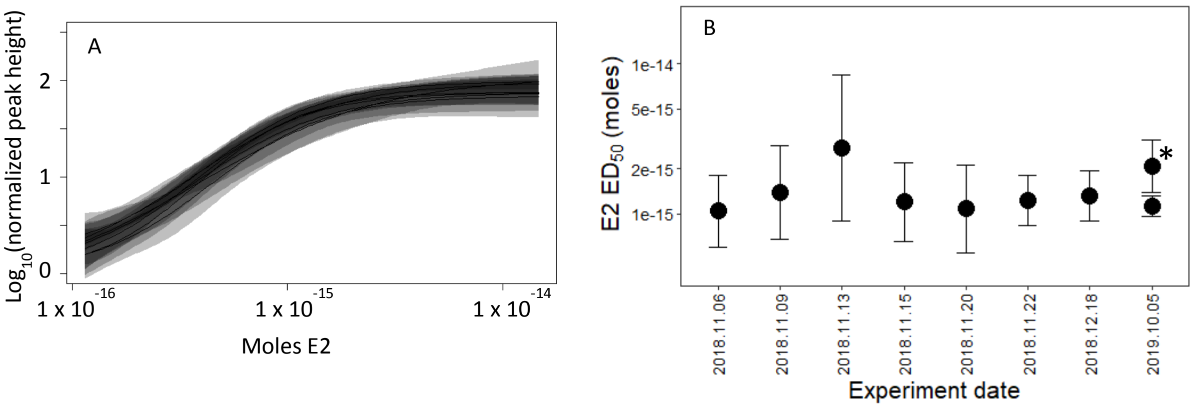

Fig. S1 Reproducibility of reference chemical, E2, in P-YES. (A) Stacked dose-response curves for E2 for seven P-YES experiments without chromatography performed in Fall 2018. Black lines are lines of best fit, grey areas are 95% confidence intervals. Overlapping dose-responses for E2, the positive control, show the reproducibility of the test. (B) The ED_{50} (moles) is reproducible over time as shown by overlapping 95% confidence intervals for eight experiments. Note log scale of y-axis. The average ED_{50} is 1.4 $x10^{-15}$ which equals 0.38 pg E2. The sensitivity of the assay was therefore consistent among data collected for publication, and after about one year. Asterisk (*) indicates an experiment performed with chromatography, demonstrating that chromatography does not universally increase the effect concentration to outside of those typically measured without chromatography

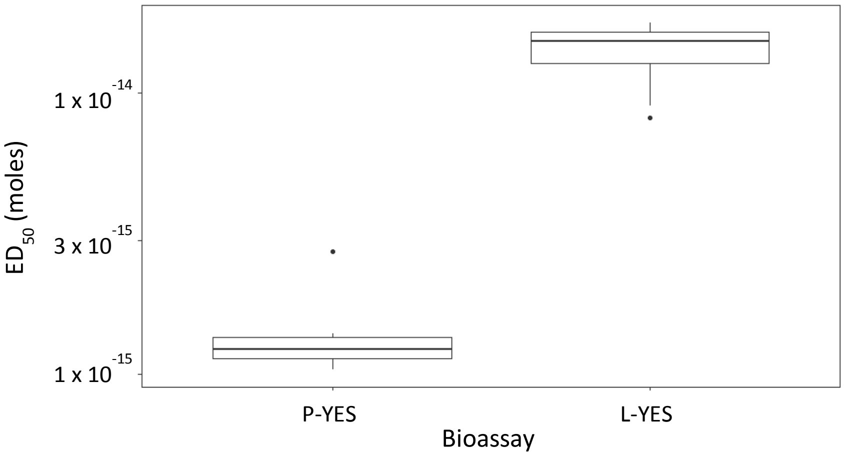

Fig. S2 Median effective doses of E2 in P-YES without chromatography compared to L-YES. $n_{P\text{-}YES} = 9$, $n_{L\text{-}YES} = 8$

Table S3 Range of doses (moles) applied in full dose-response curves of test chemicals, as determined with range finding tests

	L-YES		P-YES	
**Chemical**	Min.	Max.	Min.	Max.
2-Hydroxy-4-methoxybenzophenone	1.1×10^{-10}	1.4×10^{-08}	5.3×10^{-11}	1.4×10^{-08}
2,2'-Dihydroxy-4-methoxybenzophenone	4.7×10^{-10}	6.0×10^{-08}	2.3×10^{-10}	6.0×10^{-08}
2,4-Bis(1-methyl-1-phenylethyl)phenol	4.7×10^{-10}	6.0×10^{-08}	9.2×10^{-12}	6.0×10^{-08}
4-Nonylphenol	9.4×10^{-11}	1.2×10^{-08}	1.1×10^{-12}	7.5×10^{-09}
4-Phenylphenol	8.9×10^{-12}	1.1×10^{-09}	2.2×10^{-12}	5.7×10^{-10}
Benzene, 1,1'-(1,3-propanediyl)bis-	5.2×10^{-09}	6.6×10^{-07}	4.7×10^{-10}	1.2×10^{-07}
Benzylbutylphthalate	9.7×10^{-11}	1.2×10^{-08}	4.8×10^{-11}	1.2×10^{-08}
Bis(4-hydroxyphenyl)methane	9.7×10^{-12}	1.2×10^{-09}	4.8×10^{-12}	1.2×10^{-09}
Bisphenol A	9.8×10^{-12}	1.3×10^{-09}	3.8×10^{-13}	2.5×10^{-09}
Bisphenol B	9.7×10^{-12}	1.2×10^{-09}	4.8×10^{-12}	1.2×10^{-09}
Nonylphenolphosphite (3:1)	4.5×10^{-11}	5.7×10^{-09}	2.2×10^{-11}	5.7×10^{-09}
Phenol, 4-cyclohexyl-	9.3×10^{-13}	1.2×10^{-10}	1.8×10^{-13}	1.2×10^{-09}
Triphenyl phosphate	4.7×10^{-10}	6.1×10^{-08}	2.1×10^{-11}	9.4×10^{-08}

Table S4 Effective doses and their 95% confidence intervals at 10% (ED_{10}) and 50% (ED_{50}), and estradiol equivalency factors (EEF) calculated according to the description of Figure 2. NA: Not applicable, because the chemical was not active at the given effect level

		L-YES			P-YES		
Chemical	CAS	ED_{10}	ED_{50}	EEF	ED_{10}	ED_{50}	EEF
benzene, 1,1'-(1,3-propanediyl)bis-	1081-75-0	2.7×10^{-8} ($2.5 \times 10^{-8} – 3.0 \times 10^{-8}$)	7.7×10^{-8} ($7.3 \times 10^{-8} – 8.2 \times 10^{-8}$)	1.1×10^{-7}	4.3×10^{-8} ($9.7 \times 10^{-9} – 2.0 \times 10^{-7}$)	*NA*	1.2×10^{-8}
2,2'-dihydroxy-4-methoxybenzophenone	131-53-3	1.7×10^{-9} ($1.4 \times 10^{-9} – 2.1 \times 10^{-9}$)	*NA*	*NA*	*NA*	*NA*	*NA*
2,4-bis(1-methyl-1-phenylethyl)phenol	2772-45-4	1.2×10^{-8} ($1.1 \times 10^{-8} – 1.3 \times 10^{-8}$)	4.0×10^{-8} ($1.6 \times 10^{-8} – 9.9 \times 10^{-8}$)	4.3×10^{-7}	6.6×10^{-9} ($2.8 \times 10^{-9} – 1.5 \times 10^{-8}$)	*NA*	5.5×10^{-8}
2,4-di-tert-butylphenol	96-76-4	*NA*	*NA*	*NA*	*NA*	*NA*	*NA*
2-hydroxy-4-methoxybenzophenone	131-57-7	1.1×10^{-9} ($1.0 \times 10^{-9} – 1.2 \times 10^{-9}$)	3.7×10^{-9} ($3.3 \times 10^{-9} – 4.0 \times 10^{-9}$)	4.2×10^{-6}	1.0×10^{-10} ($7.6 \times 10^{-11} – 1.3 \times 10^{-10}$)	*NA*	3.4×10^{-6}
phenol, 4-cyclohexyl-	1131-60-8	2.3×10^{-11} ($2.0 \times 10^{-11} – 2.6 \times 10^{-11}$)	3.9×10^{-11} ($3.6 \times 10^{-11} – 4.3 \times 10^{-11}$)	2.5×10^{-4}	2.2×10^{-12} ($1.4 \times 10^{-12} – 3.5 \times 10^{-12}$)	1.4×10^{-11} ($4.7 \times 10^{-12} – 4.1 \times 10^{-11}$)	8.7×10^{-5}
4-nonylphenol	104-40-5	2.86×10^{-10} ($2.6 \times 10^{-10} – 3.1 \times 10^{-10}$)	1.1×10^{-9} ($9.3 \times 10^{-10} – 1.3 \times 10^{-9}$)	1.6×10^{-5}	1.4×10^{-11} ($9.8 \times 10^{-12} – 1.9 \times 10^{-11}$)	*NA*	2.7×10^{-5}
4-phenylphenol	92-69-3	7.5×10^{-11} ($7.0 \times 10^{-11} – 8.0 \times 10^{-11}$)	2.0×10^{-10} ($1.9 \times 10^{-10} – 2.1 \times 10^{-10}$)	9.1×10^{-5}	3.2×10^{-12} ($2.2 \times 10^{-12} – 4.7 \times 10^{-12}$)	1.8×10^{-11} ($6.0 \times 10^{-12} – 5.2 \times 10^{-11}$)	5.9×10^{-5}
4-tert-butylphenylsalicylate	87-18-3	*NA*	*NA*	*NA*	*NA*	*NA*	*NA*
bis(4-hydroxyphenyl)methane	620-92-8	1.6×10^{-10} ($1.5 \times 10^{-10} – 1.7 \times 10^{-10}$)	5.0×10^{-10} ($4.4 \times 10^{-10} – 5.7 \times 10^{-10}$)	2.9×10^{-5}	5.9×10^{-12} ($3.3 \times 10^{-12} – 1.0 \times 10^{-11}$)	5.7×10^{-11} ($1.2 \times 10^{-11} – 2.7 \times 10^{-10}$)	2.4×10^{-5}
bisphenol A	80-05-7	2.0×10^{-10} ($1.8 \times 10^{-10} – 2.2 \times 10^{-10}$)	7.5×10^{-10} ($3.1 \times 10^{-10} – 1.8 \times 10^{-9}$)	2.3×10^{-5}	9.8×10^{-12} ($8.0 \times 10^{-12} – 1.2 \times 10^{-11}$)	3.7×10^{-11} ($2.5 \times 10^{-11} – 5.6 \times 10^{-11}$)	3.3×10^{-5}
bisphenol B	77-40-7	8.4×10^{-11} ($7.8 \times 10^{-11} – 9.2 \times 10^{-11}$)	2.9×10^{-10} ($2.6 \times 10^{-10} – 3.1 \times 10^{-10}$)	5.7×10^{-5}	8.2×10^{-12} ($6.0 \times 10^{-12} – 1.1 \times 10^{-11}$)	2.4×10^{-11} ($1.1 \times 10^{-11} – 5.5 \times 10^{-11}$)	5.8×10^{-5}
butyl benzyl phthalate	85-68-7	1.3×10^{-9} ($1.0 \times 10^{-9} – 1.6 \times 10^{-9}$)	*NA*	4.7×10^{-6}	*NA*	*NA*	*NA*
butylated hydroxytoluene	128-37-0	*NA*	*NA*	*NA*	*NA*	*NA*	*NA*
diethylhexyladipate	103-23-1	*NA*	*NA*	*NA*	*NA*	*NA*	*NA*
diisobutyl phthalate	84-69-5	*NA*	*NA*	*NA*	*NA*	*NA*	*NA*

di-n-hexylphthalate	84-75-3	*NA*	*NA*	*NA*	*NA*	*NA*	*NA*
nonylphenol ethoxylate	26027-38-3	*NA*	*NA*	*NA*	*NA*	*NA*	*NA*
nonylphenylphosphite (3:1)	26523-78-4	6.2×10^{-10} (5.7×10^{-10} – 6.8×10^{-10})	2.4×10^{-9} (2.0×10^{-9} – 2.9×10^{-9})	6.9×10^{-6}	1.1×10^{-11} (3.5×10^{-12} – 3.5×10^{-11})	6.8×10^{-11} (1.9×10^{-11} – 2.5×10^{-10})	1.6×10^{-5}
triphenyl-phosphate	115-86-6	4.7×10^{-8} (8.7×10^{-9} – 2.5×10^{-7})	*NA*	1.4×10^{-7}	2.7×10^{-9} (1.9×10^{-9} – 3.7×10^{-9})	*NA*	1.4×10^{-7}

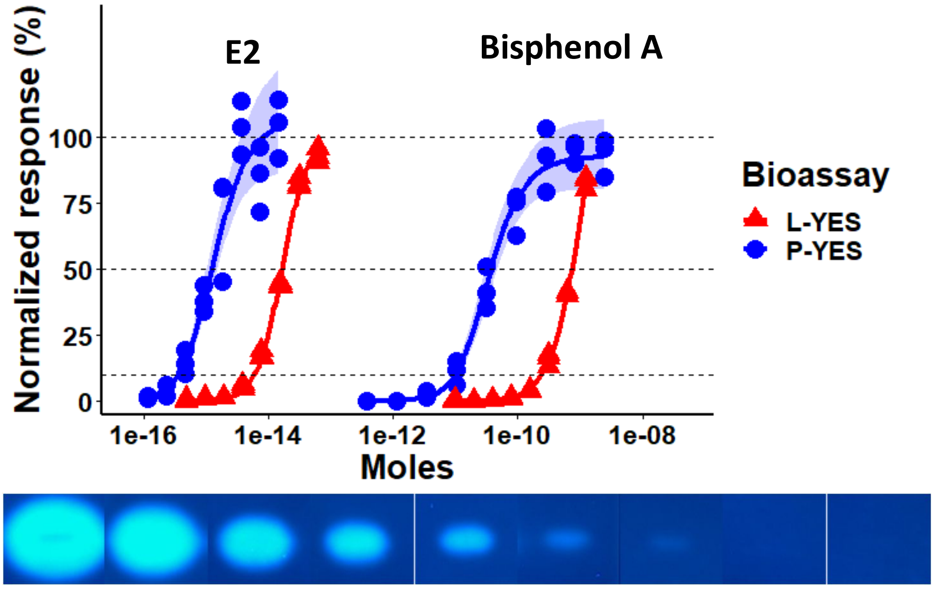
E2
Bisphenol A
Normalized response (%)
0
25
50
75
100
1e-16
1e-14
1e-12
1e-10
1e-08
Moles
Bioassay
L-YES
P-YES

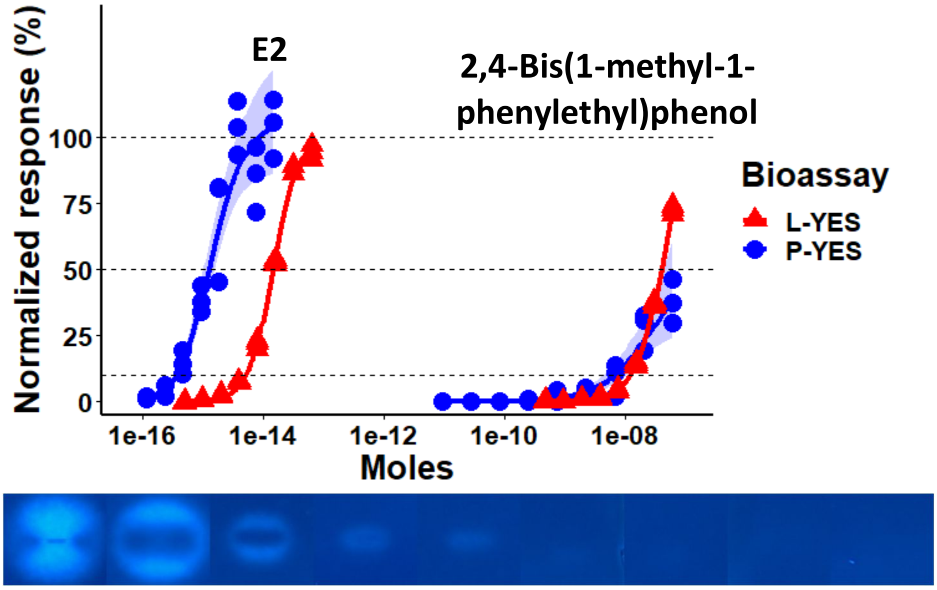
E2
2,4-Bis(1-methyl-1-phenylethyl)phenol
Normalized response (%)
0
25
50
75
100
1e-16
1e-14
1e-12
1e-10
1e-08
Moles
Bioassay
L-YES
P-YES

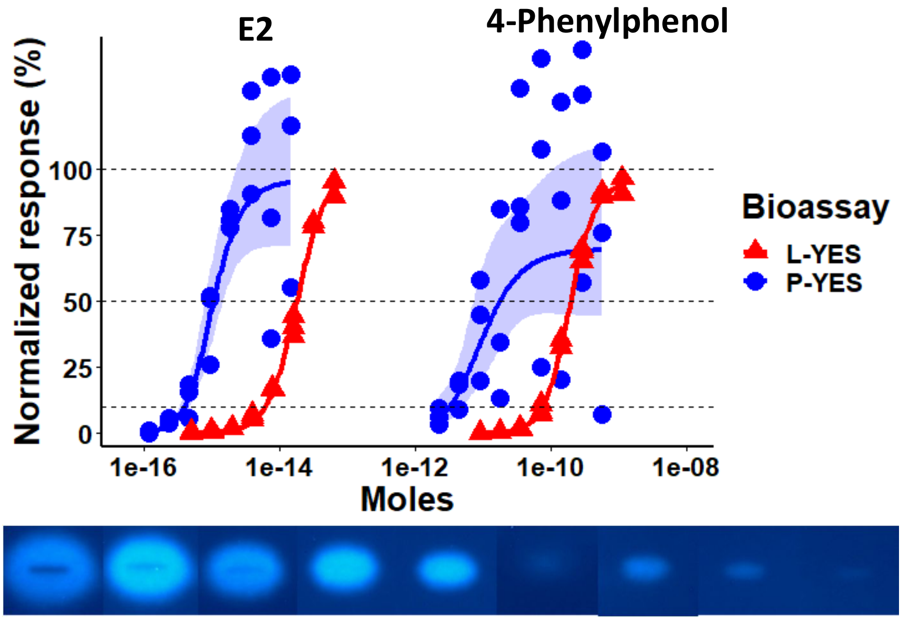
E2
4-Phenylphenol
Normalized response (%)
0
25
50
75
100
1e-16
1e-14
1e-12
1e-10
1e-08
Moles
Bioassay
L-YES
P-YES

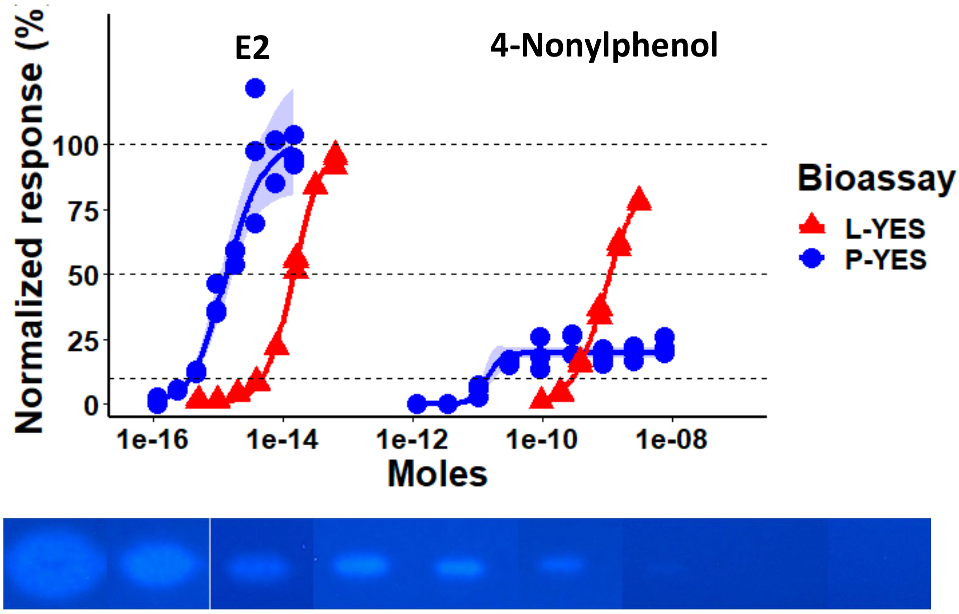
E2
4-Nonylphenol
Normalized response (%)
0
25
50
75
100
1e-16
1e-14
1e-12
1e-10
1e-08
Moles
Bioassay
L-YES
P-YES

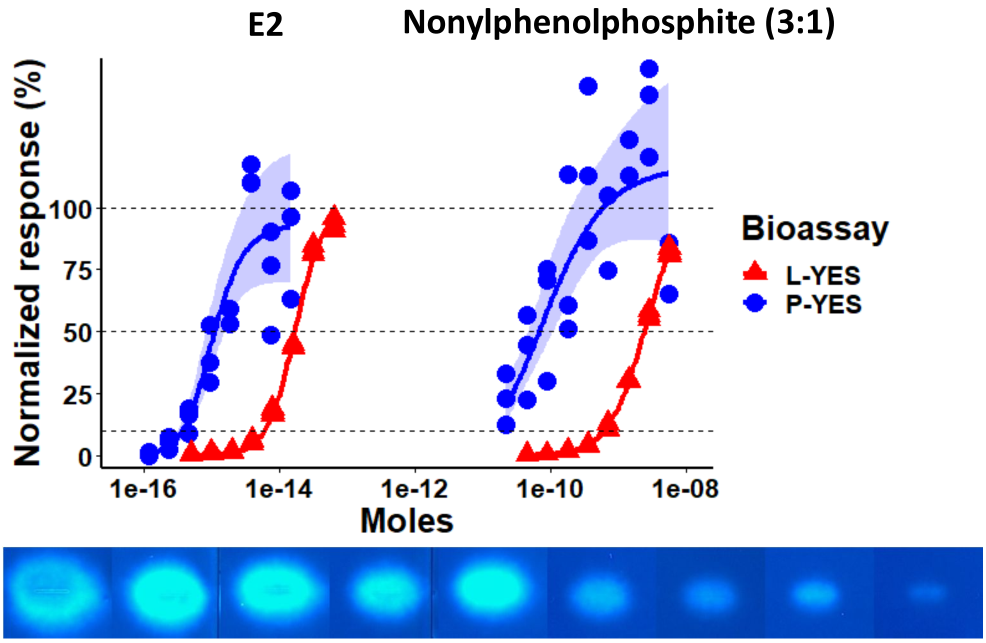
E2
Nonylphenolphosphite (3:1)
Normalized response (%)
0
25
50
75
100
1e-16
1e-14
1e-12
1e-10
1e-08
Moles
Bioassay
L-YES
P-YES

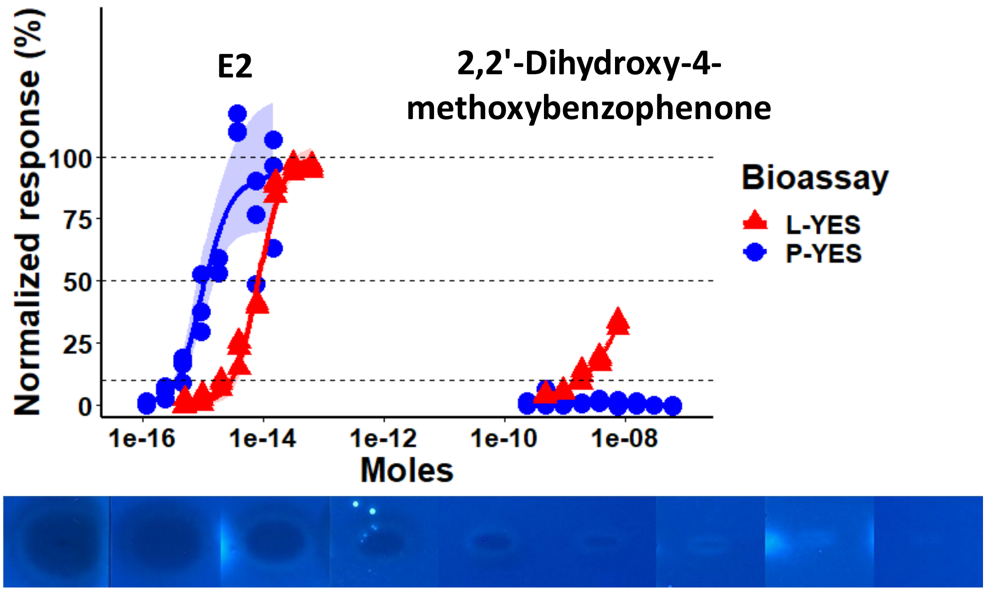

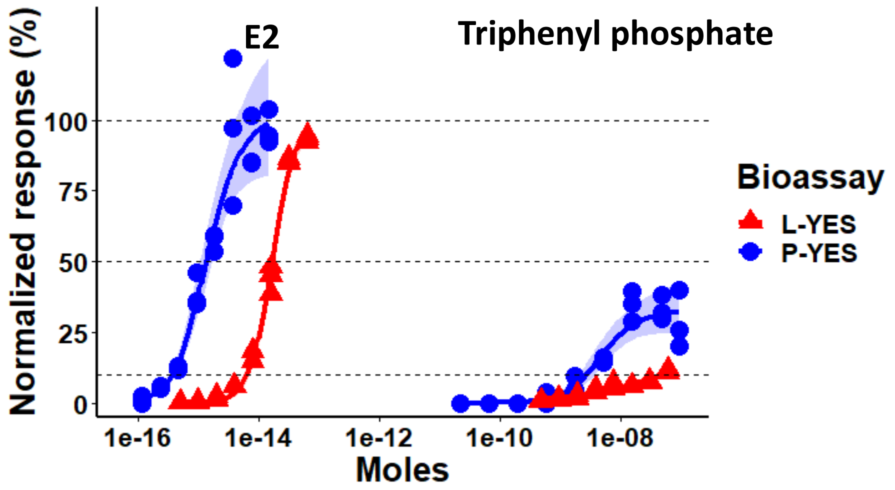

Bioassay without MUG showed fluorescence at highest two concentrations:

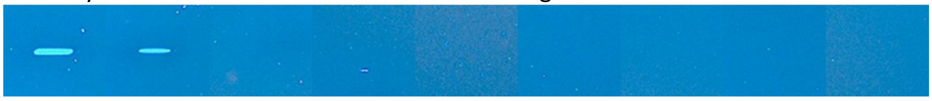

Therefore, in the bioassay with MUG, the corresponding parts of the zones were not included in the analysis by measuring the peak height only to the shoulder of the native fluorescence:

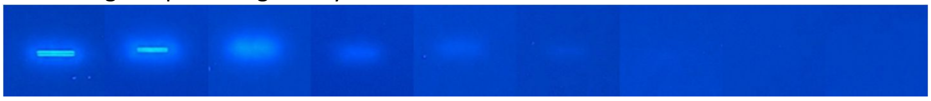

E2 **Phenol, 4-cyclohexyl**

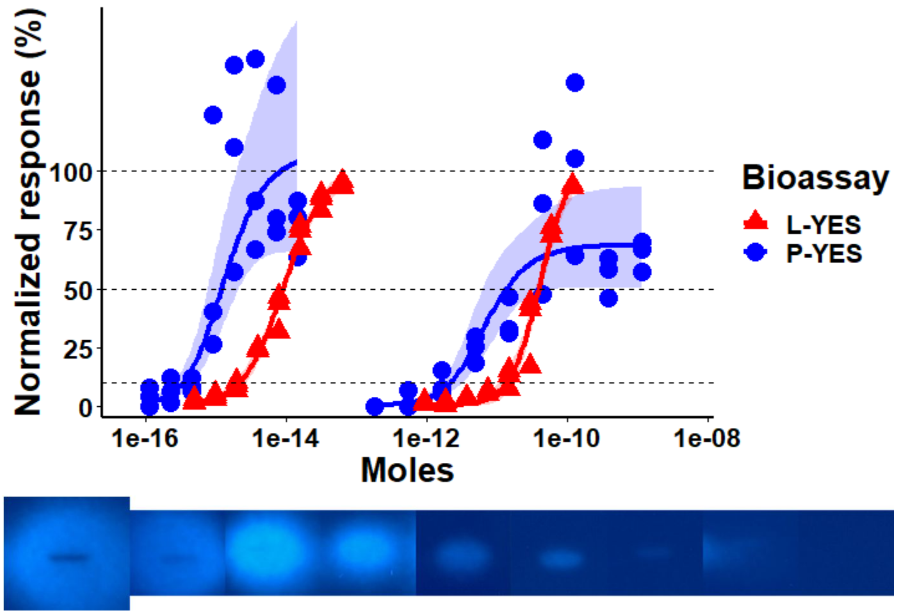
Normalized response (%)
0
25
50
75
100
1e-16
1e-14
1e-12
1e-10
1e-08
Moles
Bioassay
L-YES
P-YES

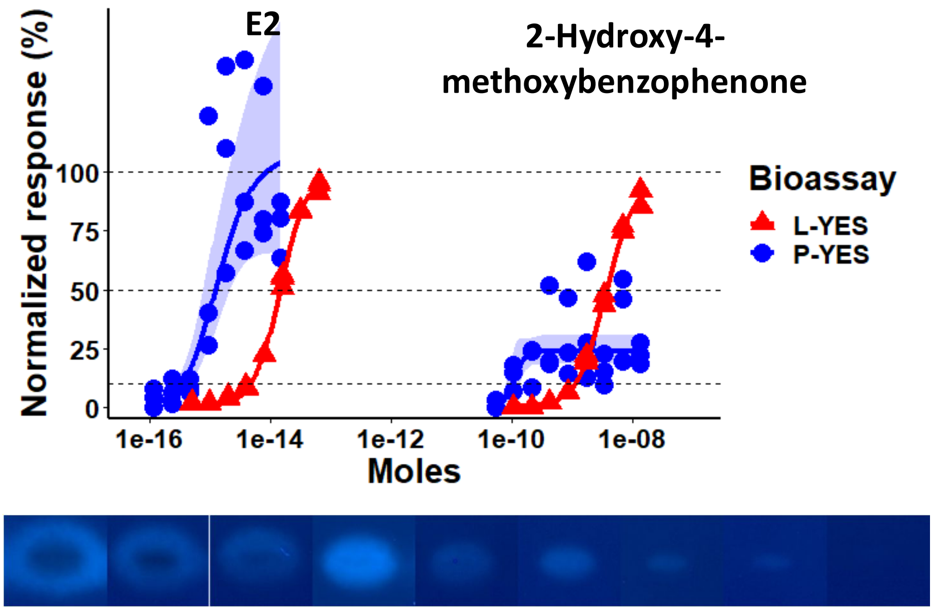
E2
2-Hydroxy-4-methoxybenzophenone
Normalized response (%)
0
25
50
75
100
1e-16
1e-14
1e-12
1e-10
1e-08
Moles
Bioassay
L-YES
P-YES

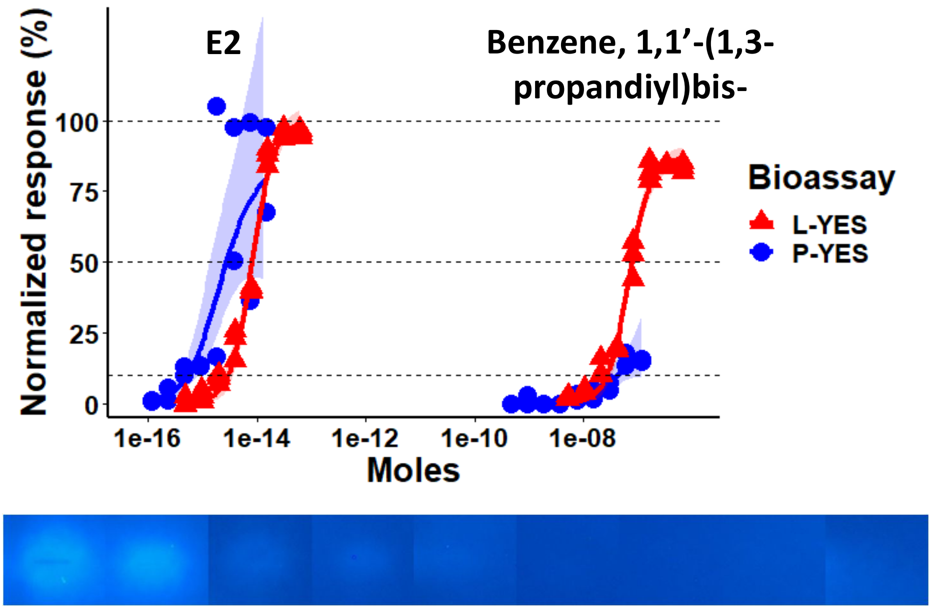
E2
Benzene, 1,1’-(1,3-propandiyl)bis-
Normalized response (%)
100
75
50
25
0
1e-16
1e-14
1e-12
1e-10
1e-08
Moles
Bioassay
L-YES
P-YES

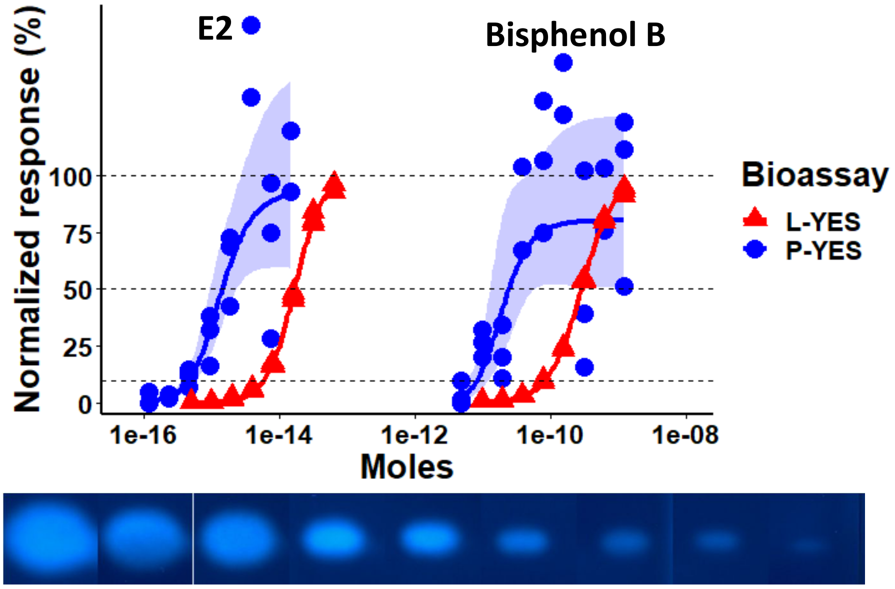
E2
Bisphenol B
Normalized response (%)
100
75
50
25
0
1e-16
1e-14
1e-12
1e-10
1e-08
Moles
Bioassay
L-YES
P-YES

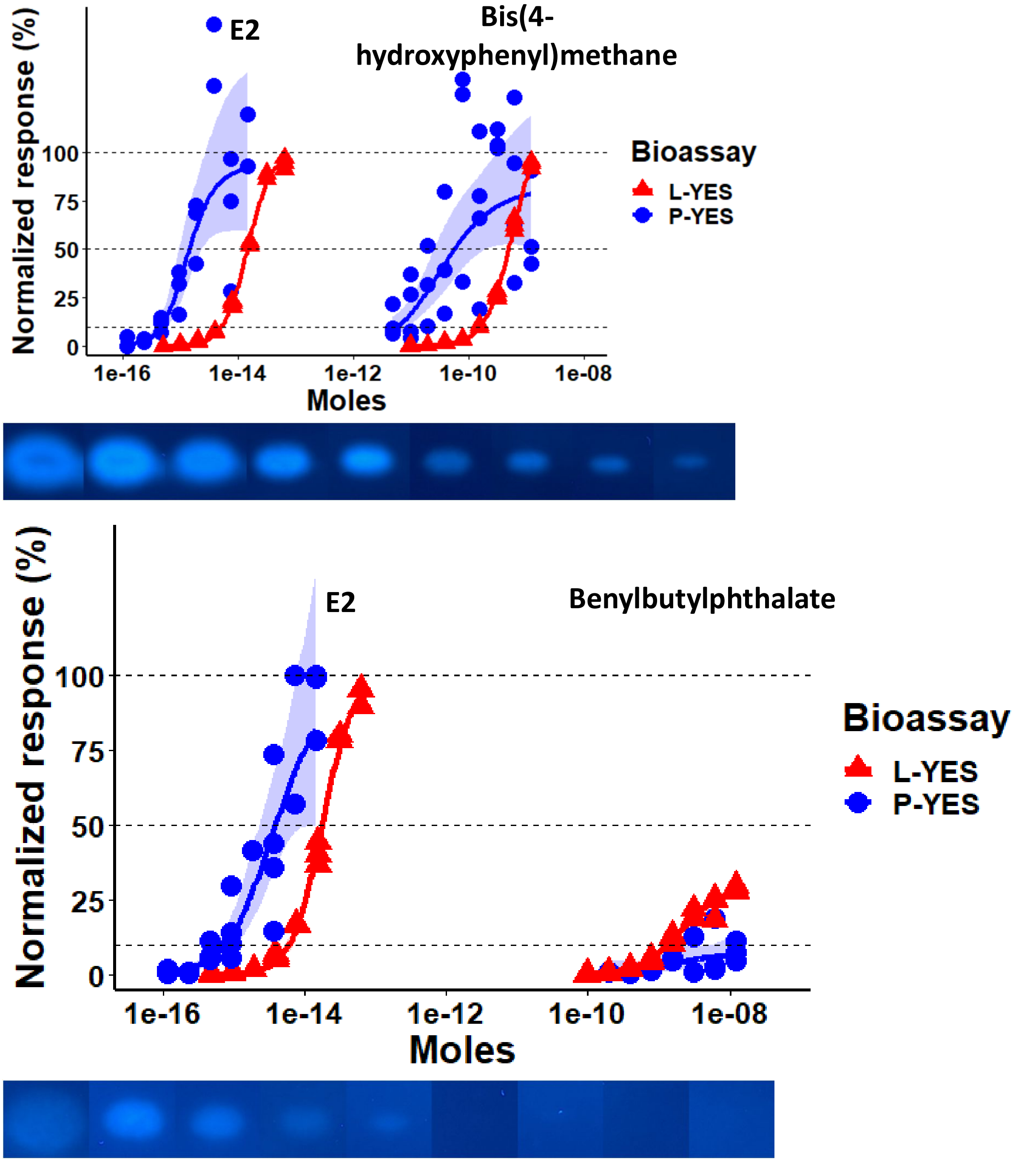

Fig. S3 Full dose-response curves of individual chemicals. P-YES modeling was performed with three replicate plates, using peak height as a metric of response. The zones were randomly assigned (randomized with Microsoft Excel) and applied to the plates so that variability on and between plates did not disproportionately affect one chemical or concentration. Example P-YES zones are shown for each level of the test chemicals below the corresponding figure, from highest dose (left) to lowest (right). We evaluated the dose-response curves of the reference chemical, E2, which was also randomly assigned to coordinates on the plates. We required the dose-response curve and ED_{50} of the reference chemical to be consistent throughout the study. Figures S1 and S2 show that these objectives were met, despite variability observed in the dose-response curves of individual chemicals, including E2. The result of log-transformed responses in dose-response modeling is to have wider confidence intervals around higher responses, which is more representative of the variability increasing with response. L-YES modeling was performed with three replicates per plate. The two

and three highest concentrations of 4-nonylphenol and 2,2'-dihydroxy-4-methoxybenzophenone, respectively, had less than 80% cell growth so were excluded from analysis

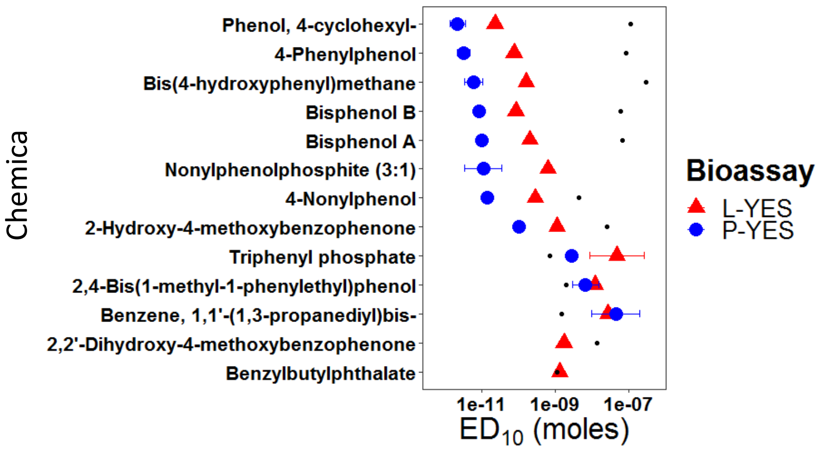

Fig. S4 Dose at 10% effect compared to water solubility. Water solubilities (black dots) of test chemicals are shown as moles at saturation in 120 µL (volume of L-YES). Specifically calculated as: water solubility (M, according to U.S.EPA Chemistry Dashboard in Table 1) times 1.2 x 10^{-6} L. Water solubility is not calculable for P-YES because the assay has no defined volume. Water solubility was not available for nonylphenylphosphite (3:1)

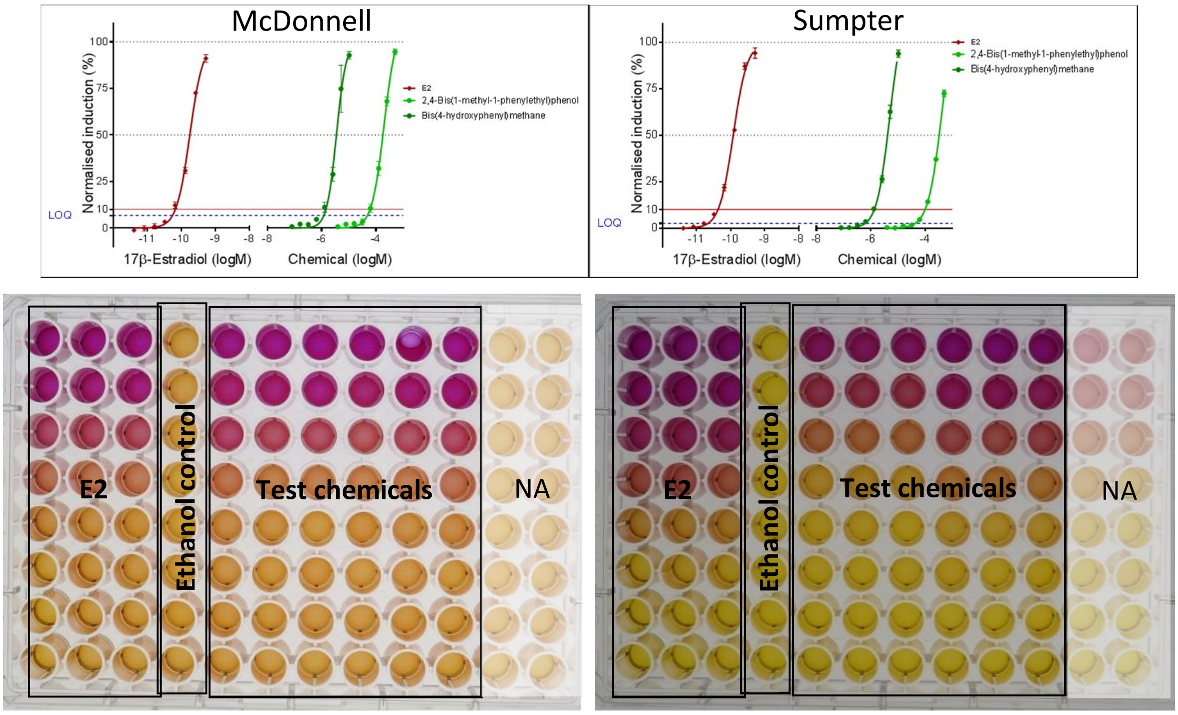

Fig. S5 Examples of McDonnell (left) and Sumpter (right) strains of yeast. Concentration-response curves are shown in the top figures for 17β-estradiol (E2, red), and Bis(4-hydroxyphenyl)methane (dark green), and 2,4-bis(1-methyl-1-phenylethyl)phenol (light green). Images of microtiter plates (bottom) show that McDonnell strain resulted in higher background than Sumpter yeast, as apparent by the darker color of the ethanol controls. Wells labeled not applicable, “NA”, were used for a different project

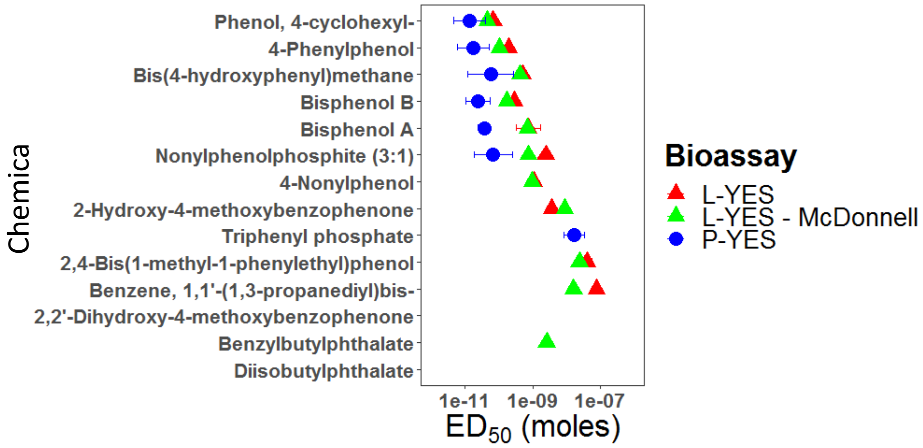

Fig. S6 Comparison of ED_{50}s derived from McDonnell yeast in L-YES with Sumpter yeast in L-YES and McDonnell yeast in P-YES. Potency of chemicals related to plastic packagingin L-YES (red triangles) and P-YES (blue circles) as shown in the main text, and L-YES with McDonnell strain yeast (green triangles). L-YES with McDonnell strain yeast is similar to L-YES with Sumpter strain yeast. The modeled top value of benzylbutylphthalate was above 50% for McDonnell, so ED_{50} was calculable where it was not calculable for Sumpter yeast L-YES

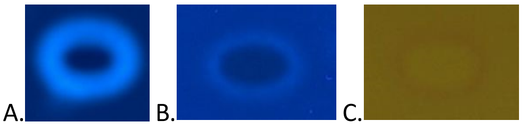

Fig. S7 Example of “halo” or “corona” effect in P-YES. (A) Bisphenol F, 1.2 x 10^{-8} moles, (B) 2,2’-dihydroxy-4-methoxybenzophene, 7.5 x 10^{-9} moles, with MUG or (C) CPRG as indicators. That the halo is apparent to similar degrees for both MUG and CPRG shows that the halo is not a result of chemical interference with UV illumination. Chemicals were applied as 6 mm, 20 µL, bands with ATS4 (CAMAG). CPRG solution: 0.4 mg/mL CPRG in *lacZ* buffer and 2 mL sprayed with Derivatizer (CAMAG)

Table S5 L-YES with CPRG and MUG. ED_{50} (mol/L) of E2 in the L-YES with 1 hour incubation of two indicator chemicals. CPRG detection performed as described in the main text. MUG detection was performed with a 30 µL aliquot of the exposed yeast into white microtiter plates. This was followed by addition of 50 µL 0.5 mg/mL MUG solution in *lacZ* buffer containing lyticase and dithiothreitol. Fluorescence was detected with a Tecan Infinite 200 microplate reader at 366 nm excitation and 440 nm emission

plate	CPRG	MUG	ratio (CPRG/MUG)
1	6.07E-11	5.33E-11	1.1
2	5.37E-11	5.01E-11	1.1
3	9.12E-11	5.35E-11	1.7
summary			
average	6.85E-11	5.23E-11	1.3
standard deviation	2.00E-11	1.93E-12	0.35

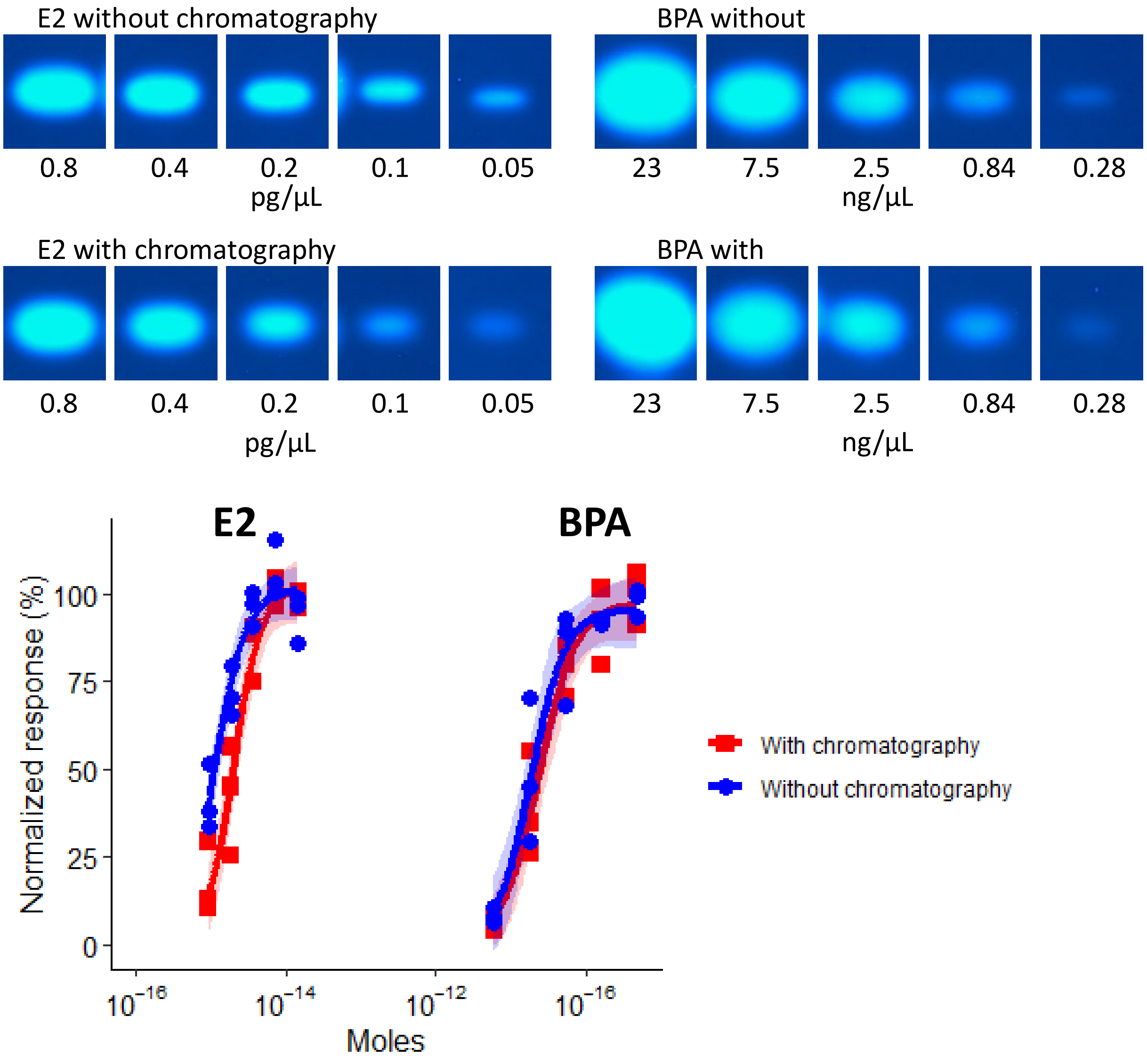

Fig. S8 Investigating the effect of chromatography. E2 and BPA were applied (5 µL) to triplicate HPTLC plates before and after chromatography. Five concentrations of E2 and BPA were applied, as randomized with Microsoft Excel, to tracks 1 – 10. Chromatography was performed as described in the main text. Then, using the same vials, the same volume was applied, as randomized with Microsoft Excel, to tracks 11 – 15 of the developed plates at 33 and 66 mm in the y direction. The P-YES was performed as described in the main text

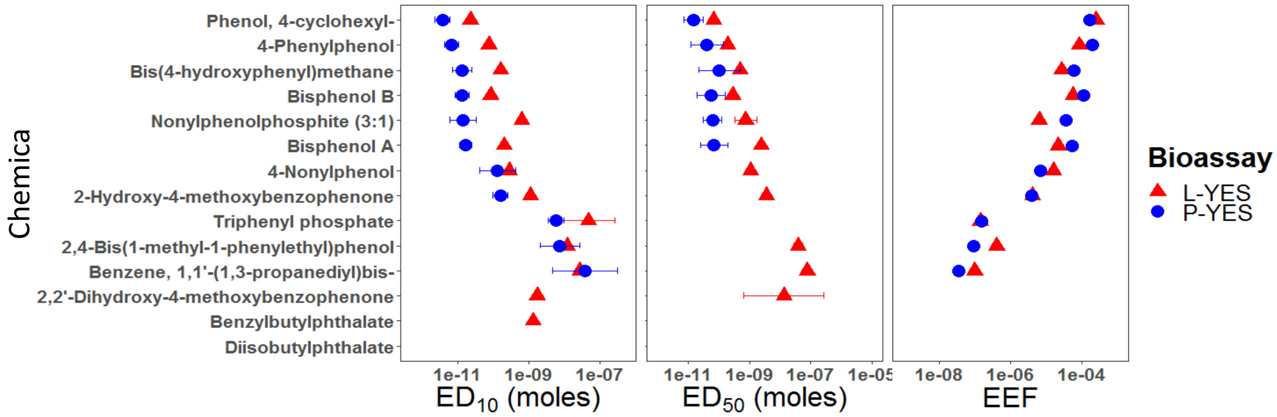

Fig. S9 Effective doses and estradiol equivalencies calculated with P-YES peak area of bioactive zone in P-YES instead of height. ED_{50}s are greater for area than for height and the error bars are larger

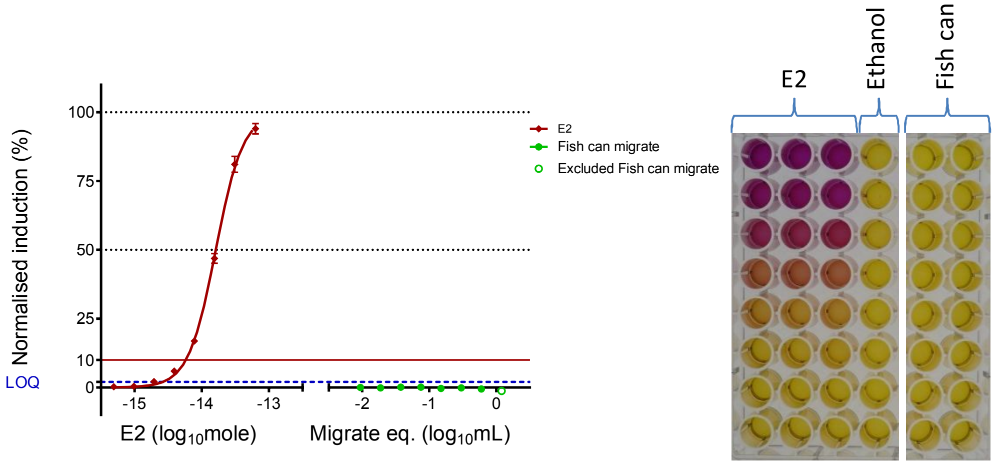

Fig. S10 Fish can migrate tested in L-YES. Sample was tested here at a maximum of 1.2 mL sample equivalents ($\log_{10}$(1.2 mL) = 0.08). Reduced cell growth was observed in highest concentration, as is indicated by an open circle. LOQ was determined as ten times the standard deviation of the ethanol control wells

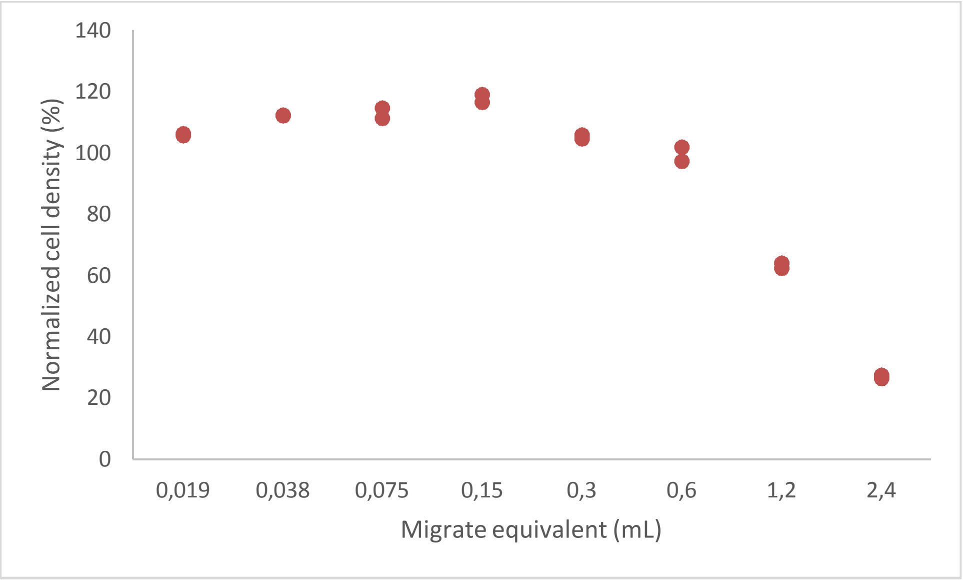

Fig. S11 Cell density in L-YES of fish can migrate, which produced no estrogenicity response. Red markers show duplicate wells. Sample was tested here in duplicate at a maximum migrate equivalent of 2.4 mL. Cell density was normalized as optical density to ethanol controls as ($OD_{600\text{-sample}}/OD_{600\text{-control}}$)

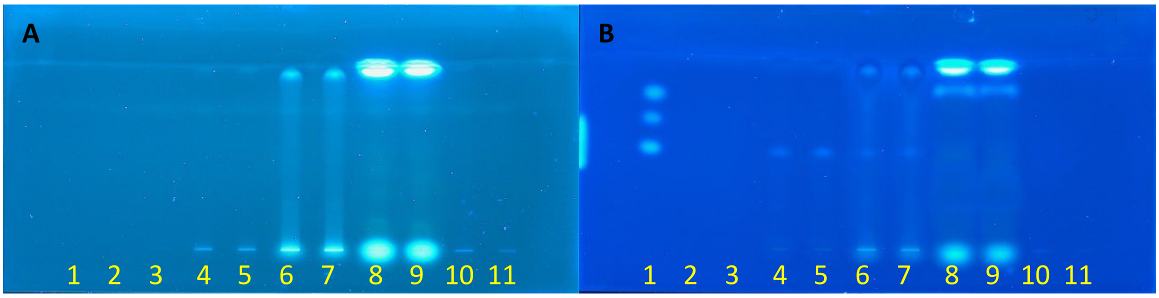

Fig. S12 Estrogen screening of food contact materials with P-YES . Fluorescence (A) after incubation with yeast but before detection of β-galactosidase with MUG (*i.e.* native fluorescence) and (B) after addition of MUG in *lacZ* buffer. From left to right the samples on the plate are (1) positive control estrogen mix, (2) solvent control, (3) sample preparation control, and duplicate migrates of (4-5) big metal cans, (6-7) small metal cans, (8-9) fish cans, and (10-11) migration negative controls. Images were collected with CAMAG Visualizer II, with illumination at 366 nm and camera duration 550 ms
